# Clinical Implications of iNOS Levels in Triple-Negative Breast Cancer Responding to Neoadjuvant Chemotherapy

**DOI:** 10.1371/journal.pone.0130286

**Published:** 2015-07-21

**Authors:** Zining Jin, Wenqian Wang, Nan Jiang, Lei Zhang, Yiming Li, Xiaoyin Xu, Shouliang Cai, Liang Wei, Xuhong Liu, Guanglei Chen, Yizhen Zhou, Cheng Liu, Zhan Li, Feng Jin, Bo Chen

**Affiliations:** Department of Breast Surgery, The First Hospital of China Medical University, Shenyang, Liaoning, China; National Cheng Kung University, TAIWAN

## Abstract

Triple-negative breast cancer is a high-risk breast cancer with poor survival rate. To date, there is a lack of targeted therapy for this type of cancer. One unique phenomenon is that inflammatory breast cancer is frequently triple negative. However, it is still ambiguous how inflammation influences triple-negative breast cancer growth and responding to chemotherapy. Herein, we investigated the levels of inflammation-associated enzyme, iNOS, in 20 triple-negative breast cancer patients’ tumors, and examined its correlation with patients’ responses to platinum-based neoadjuvant chemotherapy. Our studies showed that triple-negative breast cancer patients with attenuated iNOS levels in tumor cells after treatment showed better responses to platinum-based neoadjuvant chemotherapy than other triple-negative breast cancer patients. Our further *in vitro* studies confirmed that induction of proper levels of NO increased the resistance to cisplatin in triple-negative MDA-MB-231 cells. Our data suggest that aberrant high level of iNOS/NO are associated with less effectiveness of platinum-based neoadjuvant chemotherapy in triple-negative breast cancer. Therefore, we propose to monitor iNOS levels as a new predictor for triple-negative breast cancer patient’s response to platinum-based neoadjuvant chemotherapy. Moreover, iNOS/NO is considered as a potential target for combination therapy with platinum drugs for triple-negative breast cancer.

## Introduction

Triple-negative breast cancers (TNBCs) refer to describe those cancers which do not express oestrogen receptor (ER), progesterone receptor (PR) or overexpress human epidermal growth factor receptor 2 (Her2/neu) [1]. TNBCs account for approximately 15%-25% of all breast cancer cases [2]. Because of lack of targeted receptors, treatment options for TNBCs are restrictive. It has been reported that their phenotypic and molecular similarity to BRCA1-related breast cancers might provide implications in terms of treatment [3]. In vitro studies of BRCA1-related breast cancers appear to be particularly susceptible to platinum-based drugs chemotherapy, which could be relevant by extrapolation to the treatment of TNBCs [4, 5]. So far, there may indeed be some clinical gain with platinum-based chemotherapy for TNBCs, for instance, Garber et al. have shown a pathological complete remission rate of 21% with neoadjuvant cisplatin in a phase II study [6]. Nevertheless, the efficacy of platinum-based neoadjuvant chemotherapy in TNBCs is far from impressive, and these data have raised a heated debate of using platinum combinations in patients with TNBCs. The major molecular signaling that affects the efficacy of platinum drugs in TNBCs remains unknown. Therefore, there ought to be investigated the crucial factor responsible for TNBC patient’s response to platinum-based chemotherapy, and then we could discover new target for combination therapy, which would enhance the efficacy of platinum relevant drugs against TNBCs.

It is known that about 40% of inflammatory breast cancers (IBC) are TNBCs [7–9]. A recent study from Buchholz’s group showed that triple-negative subtype of IBC is associated with worse overall survival, distant relapse, and locoregional relapse in patients [10]. Therefore, inflammation is a crucial component of TNBC tumor microenvironment and driving force in cancer progression and resistant to apoptosis. Among various inflammatory and pro-inflammatory molecules, inducible nitric oxide synthase (iNOS) has drawn a great attention in breast cancer research. iNOS is one of three key enzymes generating nitric oxide (NO) from the amino acid L-arginine [11]. The iNOS-derived NO is a potent free radical, and could affect numerous cellular pathways. Depending on diverse cell types and intracellular concentrations, NO plays a “double impact” in the growth and progression of carcinoma cells, which promotes tumor growth and progression at lower concentration, but becomes cytotoxic at high concentration [12]. Numerous studies show that increased iNOS is associated with poor outcome and decrease survival in breast cancer, especially in the ER-negative disease [13–14]. The presence of iNOS has been observed in low-grade breast cancer, indicating its participation in the tumorigenic process [15–17]. However, other studies showed that the expression of iNOS was correlated with a higher apoptotic index in breast cancer cells [18]. To date, rare study has been conduct to explore the precise role of iNOS on tumor progression and drug response in TNBCs.

Several studies have shown that the high levels of iNOS are associated with the resistant to cisplatin in some cancer cells [19–21]. Moreover, pharmacologic attenuation of iNOS activity in those cancer cells restored their sensitivity to cisplatin. Based on the poor clinical outcome of triple-negative IBC and its modest response to platinum-based chemotherapy, we hypothesize that aberrant expression of iNOS in TNBC is associate with drug resistance and poor response to platinum-based chemotherapy.

To enhance our mechanisms of iNOS induction in TNBC and its role in tumor biology, we investigated iNOS expression in 20 TNBC patients’ tumors, and observed their responses to platinum-based neoadjuvant chemotherapy. We further examined the correlation between the iNOS expression and efficacy of neoadjuvant chemotherapy. In addition, we investigated the effects of NO on the growth of triple-negative cell line, MDA-MB-231, and its sensitivity to cisplatin. Our studies show a clinical implication of the changes of iNOS levels is correlated with poor response to platinum-based chemotherapy in TNBCs, which throw light on a new strategy to target aberrant iNOS/NO signaling for a combinational therapy with platinum drugs in TNBC patients.

## Materials and Methods

### Patient and tissue specimens

For the present study, we evaluated 20 patients who had histologically confirmed triple-negative breast cancer, received the platinum-based drugs neoadjuvant chemotherapy, and finally underwent operations in the Department of Breast Surgery of the First Hospital of China Medical University between July 2008 and April 2012. The inclusion criteria were as follows: (i) triple-negative breast cancer was histologically confirmed by diagnostic core needle biopsy; (ii) patients preoperatively were at least treated with four cycles of platinum-based neoadjuvant chemotherapy; (iii) eventual operations (lumpectomy or mastectomy) and pathological examinations were carried out; and (iv) a complete medical record was available. In all cases, tissue specimens were respectively acquired from diagnostic core needle biopsy and eventual operation. The formula for calculating tumor volume was that Volume = 4/3 x 3.1416 x (Diameter1/2) x (Diameter2/2) * (Diameter3/2).

### Ethics Statement

Ethical approval for this research was obtained from the Research Ethics Committee of China Medical University, China. All patients providing tumor tissues signed a consent form prior to diagnostic core needle biopsy and surgical removal of breast carcinoma to allow for this research to be undertaken.

### Reagents

Polyclonal rabbit anti-human iNOS antibody (dilution1:200) was obtained from Santa Cruz Biotechnology. Anti-nitrotyrosine antibody was purchased from Abcam. The 10% acrylamide gels for western blot were purchased from Bio-Rad Laboratories. DETA NONOate (nitric oxide donor) and cisplatin were purchased from Sigma. MTT Cell Proliferation Assay Kit was purchased from Life Technologies.

### Cell lines and MTT assay

MDA-MB-231 cell line was purchased from ATCC (Cat#HTB-26). MDA-MB-231 cell morphological features, proliferation, differentiation, death and migration were examined by Cell-IQ (Chip-man, Tampere, Finland). MDA-MB-231 cells were cultured in RPMI-1640 Media supplemented with 10% fetal bovine serum. About 5 × 10^4^ cells per well were plated on 24-well plates and incubated for 24 hrs. After then, cells were treated with either DETA NONOate at 1, 5, 15, and 30 μM with or without the addition of 0.5 or 2.5 μM cisplatin respectively. Each group had a blank control (1x PBS). The plates were transferred to 37°C incubator for further culture. After 72 hrs, cells were collected for protein analysis and MTT Assay. MTT assay was carried out with MTT Cell Proliferation Assay Kit based on the manufacture protocol (Life Technologies). All assays were performed in triplicate and repeated thrice.

### Immunohistochemistry

We fixed thin slices of tumor tissue of all cases received in our histopathology unit in 4% formaldehyde solution (pH 7.0) for periods not exceeding 24 h. The tissues were processed routinely for paraffin embedding, and 4 μm-thick sections were cut and placed on glass slides coated with 3-aminopropyl triethoxysilane for immunohistochemistry. Tissue samples were stained with hematoxylin and eosin to determine histological type and grade of tumors.

All cases of breast tumor tissues were cut at a thickness of 4 μm using a cryostat. The sections were mounted on microscope slides, air-dried and then fixed in a mixture of 50% acetone and 50% methanol. The sections were then de-waxed with xylene, gradually hydrated with gradient alcohol, and washed with PBS. Sections were incubated overnight at 4 degree with the primary antibody after 3%. H2O2 incubating 10 min at room temperature. Following washings with PBS, sections were incubated for 20 min at 37 degree in the Polymer Helper (PV-9000; Zhongshan Goldenbridge Ltd.). Following washing, the secondary antibody (Poly Peroxidase-anti-mouse/rabbit immunoglobulin; Zhongshan Goldenbridge Ltd.) was then applied to the sections for 30 min at 37 degree. The immunoreactive products were visualized by catalysis of 3, 3-diaminobenzidine (DAB), followng extensive washings. Sections were then counterstained in Gill's Hematoxylin and dehydrated in ascending grades of methanol before clearing in xylene, and mounting under a coverslip.

To score iNOS as immuno-positive staining: the positive cells are shown as yellow or/and a yellow to brown color of the cytoplasm.

iNOS expression was classified semi-quantitatively according to the following criteria:(1) 0 if < 10% of neoplastic cells discretely expressed iNOS in their cytoplasm; 1+ if ≥ 10 and < 50% of morphologically unequivocal neoplastic cells discretely expressed iNOS in their cytoplasm; and, 2+ if ≥ 50% of morphologically unequivocal neoplastic cells discretely expressed iNOS in their cytoplasm. (2) staining intensity divided achromatic colour, light yellow, and yellow and brown, as 0, 1, and 2 score. Two phase score multiplication results as 2 and more than 2 were considered positive.

### Western blot

Protein samples (50 μg) were mixed with 1/4 volume of SDS sample buffer, boiled for 5 min., and then separated through 10% SDS-PAGE gels. After electrophoresis, proteins were transferred to nylon membranes by electrophoretic transfer. Membranes were blocked in 5% milk for 1 hr, rinsed and incubated with primary antibodies in TBS diluted at 1:1000 at 4°C overnight. Primary antibody was then removed by washing in TBS-tween thrice, and labelled by incubating with 0.1 mg/ml peroxidase-labelled secondary antibodies against the mouse and rabbit for 1 hr. Bands were visualized by electrochemiluminescence (ECL) and exposed to X-ray film following washing thrice in TBS-tween.

### Statistical analysis

All data were analyzed by independent-Sample t-Test. Values between groups were compared by the Student's t-test, after ANOVA analyses. All experiments were repeated and P < 0.05 was considered statistically significant.

## Results

### Clinical and histological characteristics and the expression of iNOS protein in clinical specimens of TNBC patients before and after treatment

**Fig 1:**
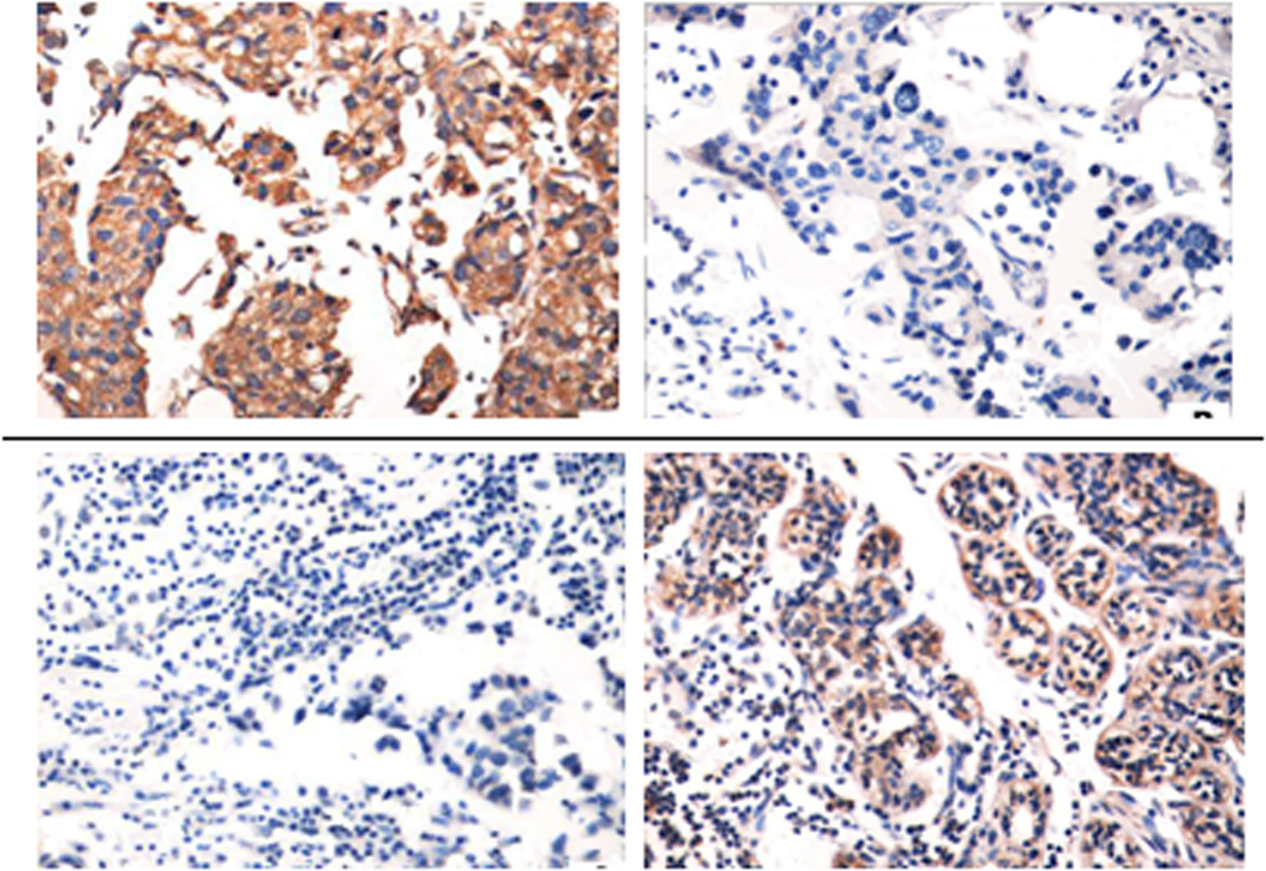
Detection of iNOS protein levels in TNBC tissues by immunohistochemistry. Top panel: demonstration of iNOS levels in patient’s tumor changing from positive to negative after treatment in IDC. Bottom panel: demonstration of iNOS levels in patient’s tumor changing from negative to positive after treatment in ILC.

**Fig 2:**
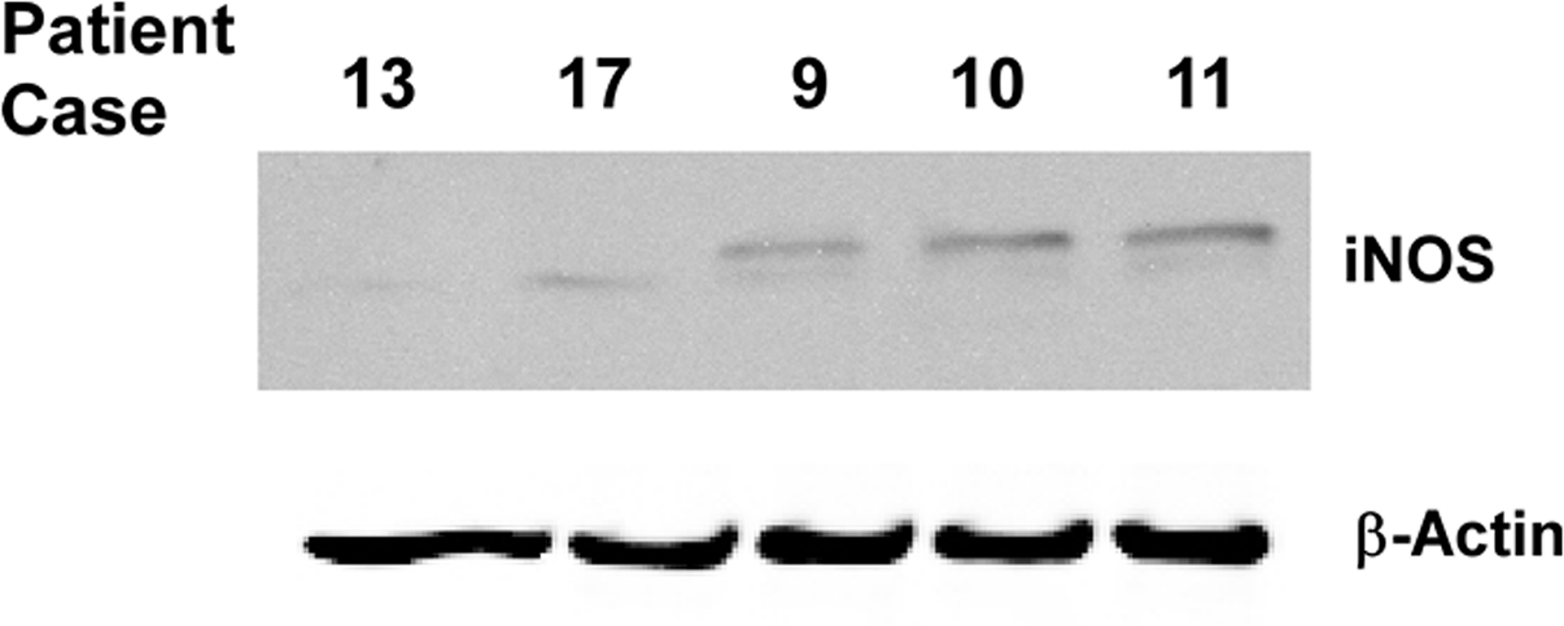
Analysis of iNOS protein levels in TNBC patients by immunoblotting.

The clinical profiles of TNBC patients in this study are shown in Table 1. By employing IHC, we examined the expression of iNOS protein in human tumor tissues from 20 TNBC patients before and after platinum-based drugs neoadjuvant chemotherapy. Immunohistochemical examination showed iNOS was mainly located in the cytoplasm of TNBC cell (Fig 1). Furthermore, the expression of iNOS protein in patient’s tumor tissues was validated by immunoblotting (Fig 2).

**Table 1:** Clinical and histological characteristics of TNBC patients before and after neoadjuvant chemotherapy. (n = 20).

No.		Diagnosis	Endoscopic expression	Tumor diameter(cm)
			(before and after neoadjuvant therapy)	(before and after neoadjuvant therapy)
**PR**				
1	Before	IDC	positive	4.0*4.0*4.0
	After	IDC	positive(2+→1+)	2.0*2.0*2.0
2	Before	IDC	positive	4.0*4.0*4.0
	After	IDC	negative	1.0*0.3*0.7
3	Before	IDC	positive	4.3*4.3*2.2
	After	IDC	positive(unchanged)	2.8*2.0*1.5
4	Before	IDC	positive	7.9*6.0*3.2
	After	IDC	negative	3.0*2.5*2.0
5	Before	IDC	positive	6.0*6.0*6.0
	After	IDC	negative	4.0*3.8*3.0
6	Before	IDC	positive	3.0*3.0*3.0
	After	IDC	negative	2.0*1.5*1.5
7	Before	ILC	positive	3.8*2.1*4.1
	After	IDC	positive(2+→1+)	2.0*1.7*1.5
8	Before	IDC	positive	9.0*7.0*6.0
	After	IDC	positive(unchanged)	5.5*4.0*4.5
9	Before	IDC	positive	3.5*3.5*3.0
	After	IDC	positive(1+→2+)	2.5*2.0*2.0
10	Before	IDC	positive	6.0*4.0*4.0
	After	IDC	negative	2.0*2.0*2.0
11	Before	IDC	positive	6.0*5.0*4.0
	After	IDC	positive(unchanged)	4.0*3.0*4.0
12	Before	ILC	positive	10.0*10.0*10.0
	After	IDC	negative	6.4*5.5*5.0
13	Before	ILC	negative	6.9*5.7*5.0
	After	IDC	positive	3.2*3.0*3.5
14	Before	IDC	positive	12.0*12.0*6.5
	After	IDC	positive(2+→1+)	3.0*3.0*3.0
15	Before	IDC	positive	20.0*15.0*15.0
	After	IDC	negative	5.0*4.0*4.0
16	Before	IDC	positive	7.0*6.0*6.0
	After	IDC	negative	2.0*1.0*1.0
**SD**				
17	Before	IDC	negative	5.0*5.0*5.0
	After	IDC	positive	4.0*4.0*4.0
18	Before	IDC	positive	5.0*4.0*4.0
	After	IDC	positive(unchanged)	4.0*3.0*3.0
19	Before	IDC	positive	8.0*7.0*6.0
	After	IDC	positive(unchanged)	7.0*6.0*6.0
20	Before	IDC	positive	6.7*4.5*4.0
	After	IDC	positive(1+→2+)	4.8*4.0*4.0

ILC: Invasive Lobular Carcinoma, IDC: Invasive Ductal Carcinoma, PR: partial response, and SD: stable disease.

Before neoadjuvant chemotherapy, we observed that positive expression of iNOS protein was 18/20. In this positive expression, iNOS was scored as grade 1+ and 2+ according to standardized criteria. Only two patients’ tumor specimens are negative expression to anti-iNOS IHC staining. This data suggest that most of TNBC tumor cells express iNOS, which may be a common inflammatory characteristic for TNBC. After neoadjuvant chemotherapy, the levels of iNOS in eight patients were changed from positive to negative. Moreover, for three patients, the iNOS levels in tumors were changed from grade 2+ to grade 1+. Interestingly, for those two patients, whose tumors were negative to iNOS before neoadjuvant chemotherapy, their tumors became positive to iNOS. There are also two patients’ tumors show an increase of iNOS levels from grade 1+ to grade 2+. The iNOS expression remained unchanged in five patients (Table 1). Therefore, the iNOS levels have been decreased in 55% of patients after neoadjuvant chemotherapy.

### Correlation between iNOS Protein Expression Changes after treatment and the responses to neoadjuvant chemotherapy in TNBC patients

**Fig 3:**
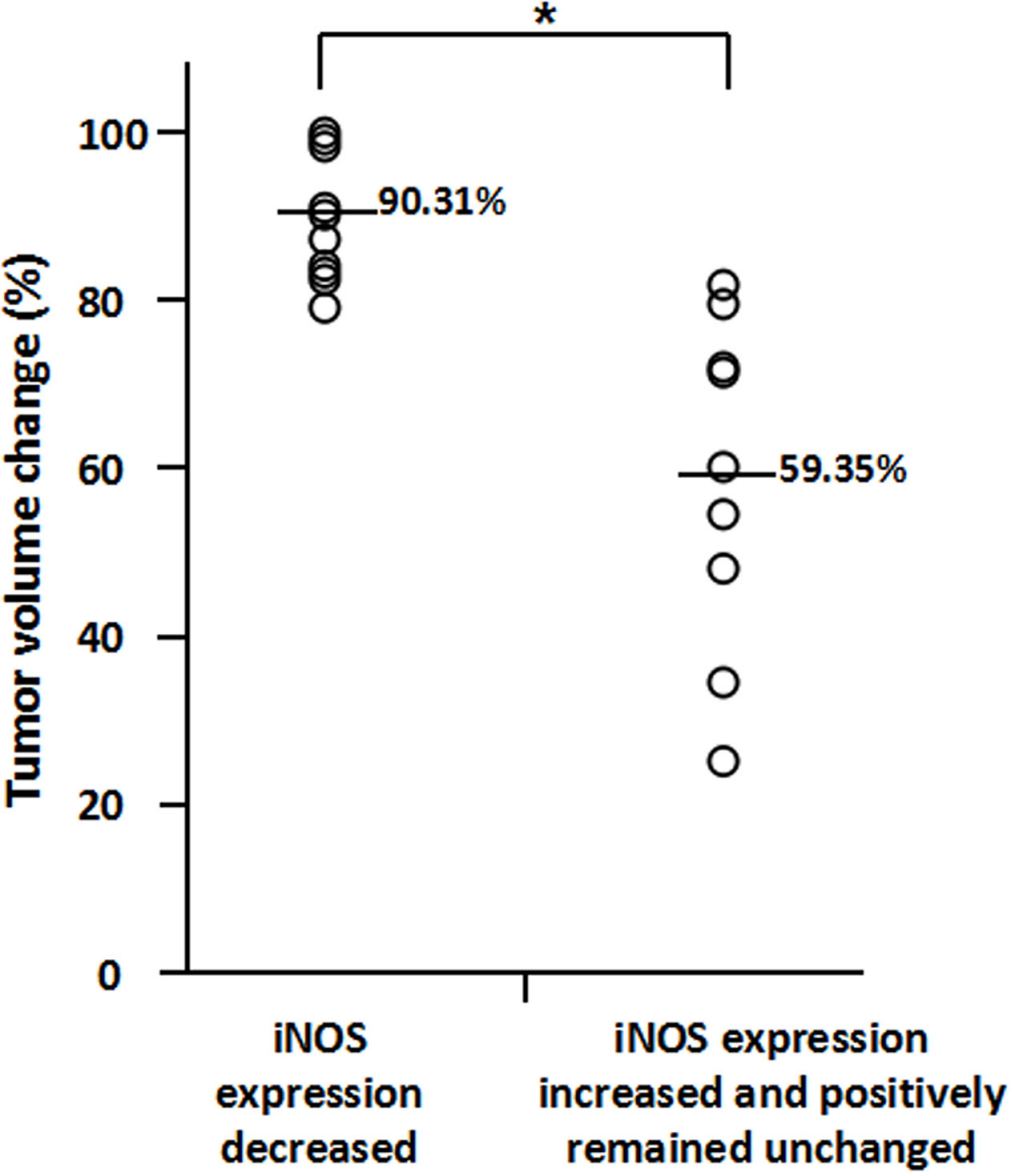
Comparison of tumor volume reduction in TNBC patients between iNOS expression decreased group and iNOS expression increased and positively remained unchanged group after neoadjuvant chemotherapy. Asterisk indicates p<0.01.

**Fig 4:**
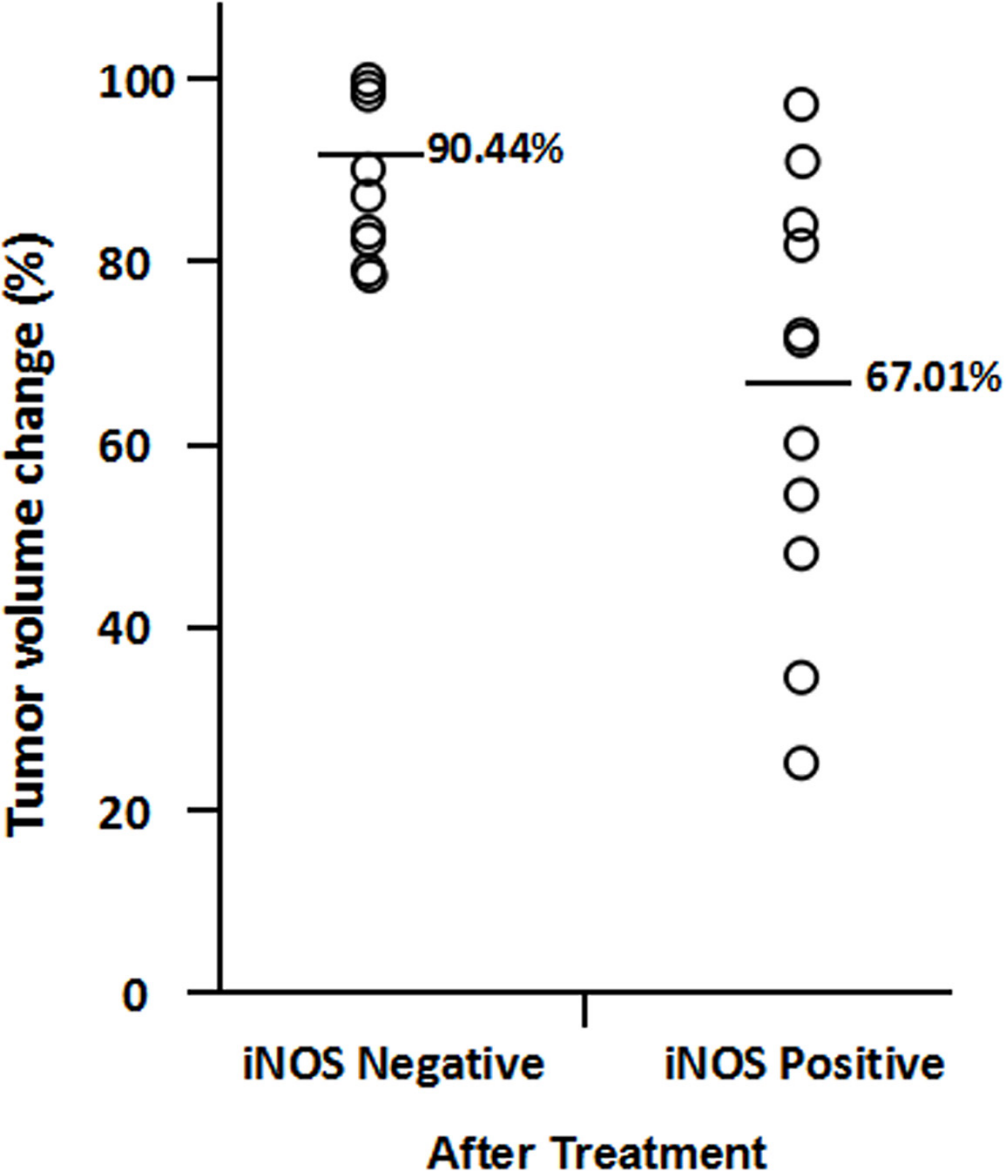
Comparison of tumor volume reduction in TNBC patients between positive expression of iNOS and negative expression of iNOS after neoadjuvant chemotherapy.

We assessed whether the change of iNOS levels after treatment was a significant predictor of TNBC patient’s response to neoadjuvant chemotherapy. Among the TNBC patients with attenuated iNOS expression after treatment (from positive to negative and from grade 2+ to grade 1+), the average of tumor volume reduction was 90.31% (Fig 3). However, the TNBC patients with increased iNOS expression (from negative to positive and from grade 1+ to 2+) and unchanged iNOS expression showed an average of 59.35% tumor reduction, which is significantly lower than attenuated iNOS expression group (p< 0.01) (Fig 3). As demonstrated in Fig 4, the TNBC patients with negative expression of iNOS after treatment showed the average of 90.44% tumor reduction, which is higher than the average of 67.01% tumor reduction in TNBC patients with positive expression of iNOS after treatment. It indicated that TNBC patients with attenuated iNOS expression after treatment are more sensitive to platinum-based neoadjuvant chemotherapy.

### The effects of NO on the growth of TNBC cell line and on resistance to cisplatin

**Fig 5:**
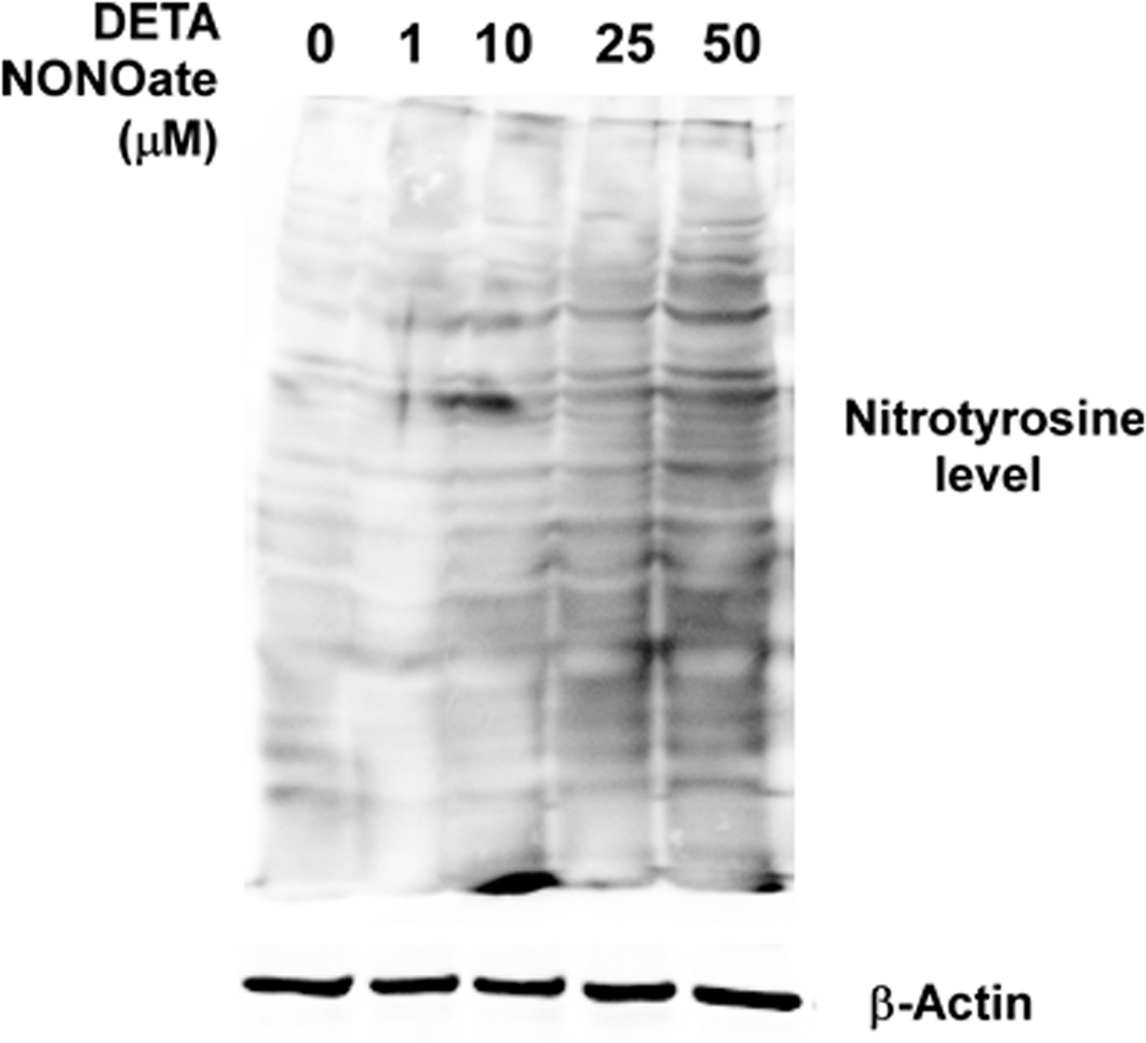
Western blot demonstration of increase nitrotyrosine levels in MDA-MB-231 cells after treatmented with NO donor.

**Fig 6:**
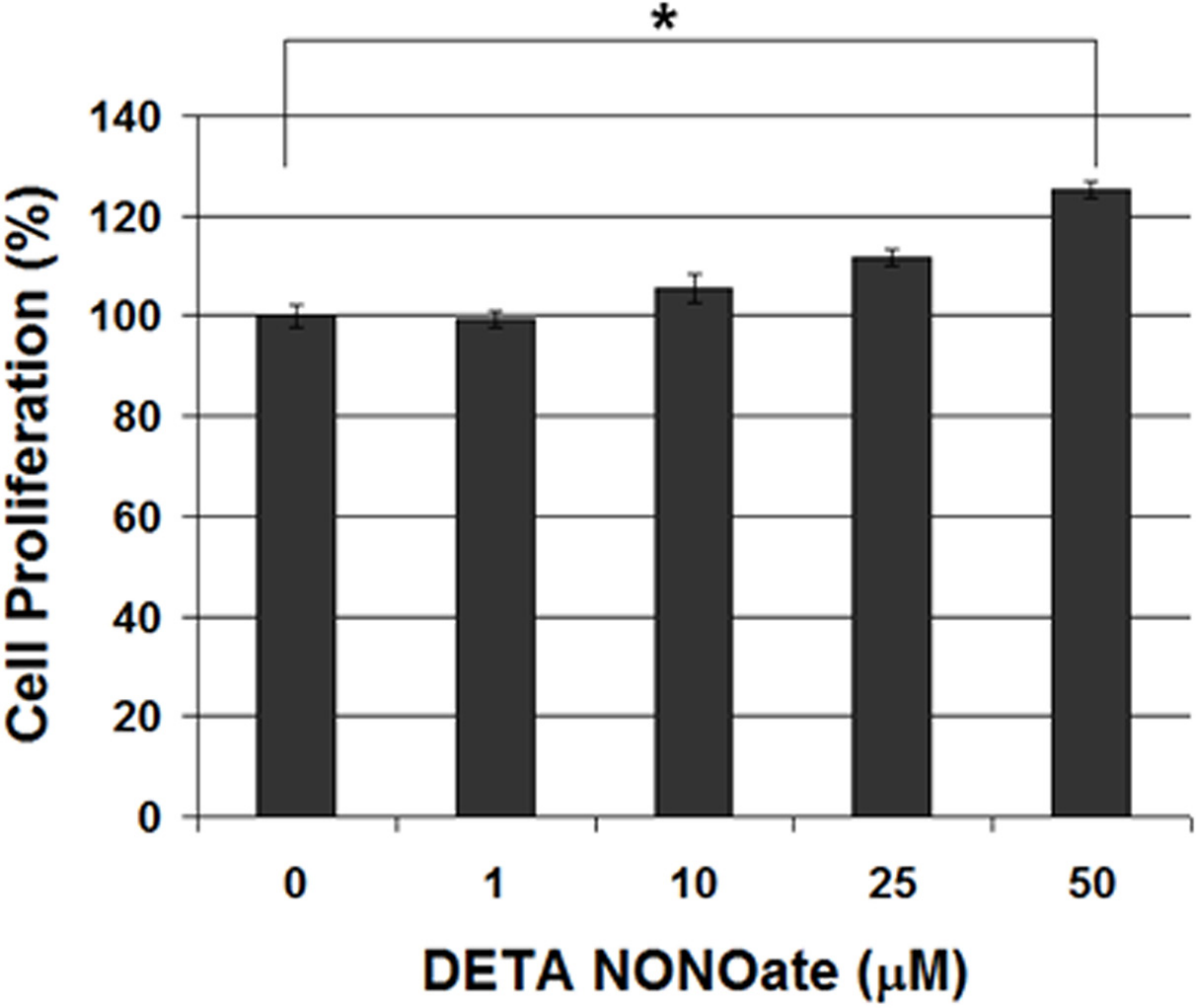
Treatment of MDA-MB-231 cells with DETA NONOate results in a increase of growth, MTT assay was performed after 72-hour treatment.

In order to explore the role of NO on TNBC cells resistance to cisplatin, we treated triple-negative MDA-MB-231 cells with NO donor, DETA NONOate. The main concentrations of DETA NONOate used in our studies were lower than 50 μM, which were designed to exogenously provide MDA-MB-231 cells with growth-supporting concentrations of NO mimicking the chronical inflammatory intra-cellular NO produced by iNOS in cancer cells [13]. One of important downstream products for NO in cells is the abduction of NO on protein tyrosine residue, which is termed nitrotyrosine. As shown in Fig 5, the addition of DETA NONOate increased the levels of nitrotyrosine in MDA-MB-231 cells, confirming the increase of intra-cellular NO levels. After a 72-hr culture with DETA NONOate, no any significant downregulation of cell proliferation among all drug concentrations. Moreover, 15 and 30 μM of DETA NONOate significantly increase about 10~25% of cells proliferation compared to the cells without any treatment (Fig 6). It suggests that substantial high levels of NO promote the growth of breast cancer cells. It is known that high levels of NO are cytotoxic. We also observed that 100 μM of DETA NONOate inhibited about 40% of cell growth of MDA-MB-231 cells, which is different from the growth-supporting conditions applied in our studies.

**Fig 7:**
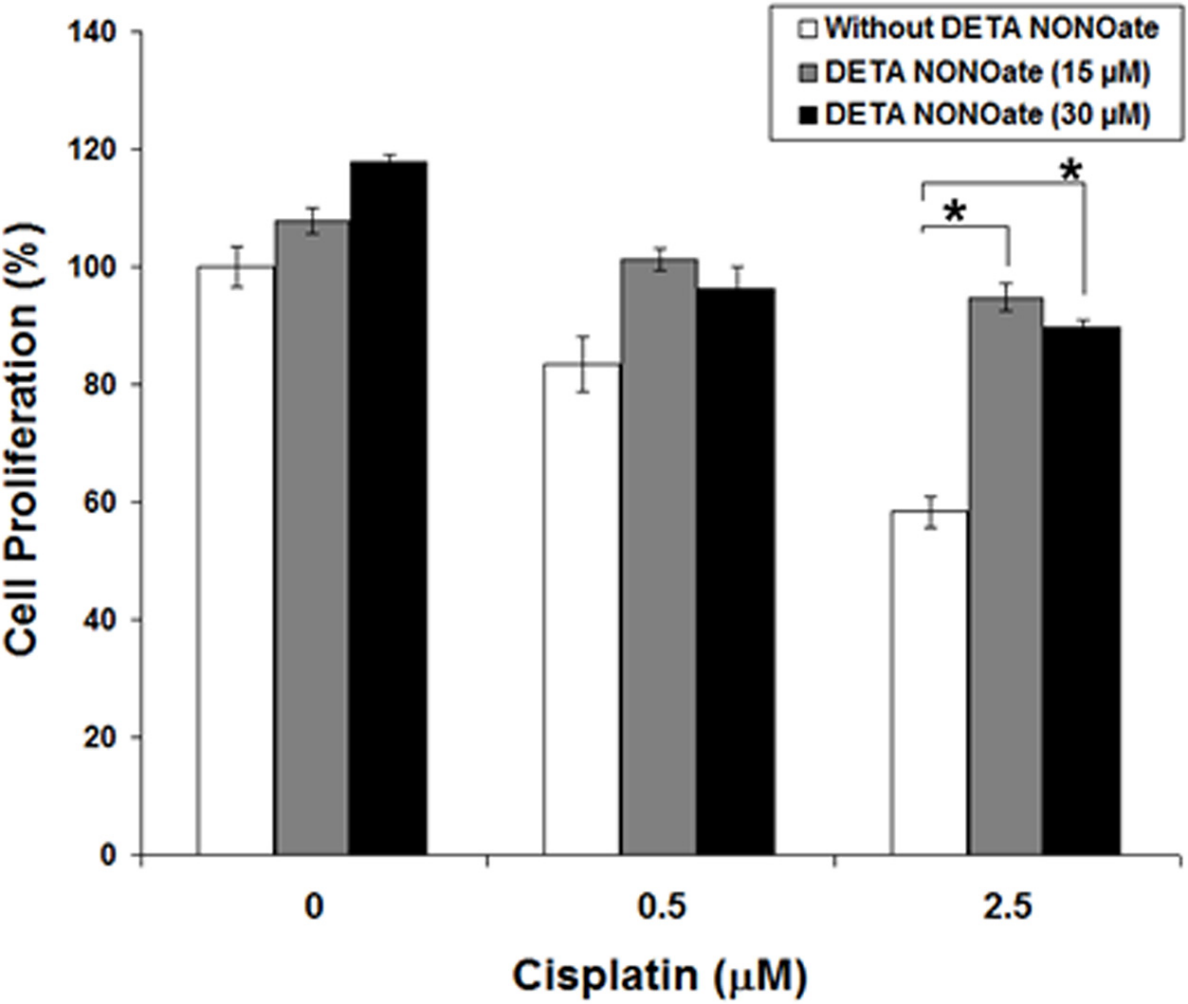
The presence of DETA NONOate reverse the inhibitory effect of cisplatin on MDA-MB-231 cells. Asterisk indicates p<0.01.

We next determine whether the presence of substantial high levels of NO translated to enhanced resistance of breast cancer cells to traditional apoptosis-induced reagent, such as cisplatin. Cisplatin, applied to the MDA-MB-231 cells at concentrations ranging from 0.5 μM to 2.5 μM, was found to efficiently downregulate cell proliferation about 15~45% in a dose-dependent manner compared to untreated controls (Fig 7). However, the growth inhibitory effects of cisplatin was markedly diminished in MDA-MB-231 cells treated with 15 or 30 μM of DETA NONOate at the same time (Fig 7). Taken together, our data suggest that endogenous increase of NO lead to the increase of resistance to apoptosis-induced reagent in breast cancer cells.

## Discussion

TNBC is a high-risk breast cancer on account of younger age, poorly differentiated histologies and poorer survival than those with other breast cancers [22, 23]. No specific systemic regimen is recommended for the treatment of these patients because of the lack of the benefit of targeted therapies. Interestingly, there was the observation that cell lines of the BRCA1 phenotype and by extrapolation TNBC cell lines generally appeared to be highly sensitive to platinum salts. However, the clinical activity of cisplatin and carboplatin against unselected breast cancer was found many years ago to be modest with an overall response rate of 32%-54% and clinical experience with them is therefore limited[24–26]. Therefore, the searching for secondary drug targets to combine with platinum drugs for the treatment in TNBC patients become a hot area for pharmaceutical studies in TNBCs.

In order to identify new drug targets in TNBCs, it is urgent to determine new clinical characteristics and to discover new biomarkers. Because of its high rate of metastasis and poor prognosis, IBC and non-IBC have attracted great attention in differing clinical behaviors between different subtypes of TNBCs. Notably, about 40% of IBCs are triple-negative, indicating inflammatory characteristics may play crucial roles in the growth and progression of TNBCs. Indeed, recent study from Buchholz’s group show that triple-negative disease is associated with worse overall survival, distant relapse, and locoregional relapse in IBC patients. Moreover, multiple inflammatory signaling pathways involving supporting tumor growth and metastasis are considered as attractive new drug targets for TNBCs.

As an approach to integrate the characteristics of IBC and TNBCs together to identify new drug targets, our study focus on the critical inflammatory enzyme iNOS. iNOS and its product NO are well-known oxidative signaling to support cancer cells growth in multiple tumors. The accurate effects of iNOS/NO on breast cancer growth are complex and remain largely unclear. Several studies have associated a higher iNOS expression and activity with higher malign carcinomas and increase angiogenesis, indicating the tumor-promoting role to iNOS/NO [12]. Conversely, a number of studies showed conflicting results. It was found that the level of iNOS expression is inversely correlated to tumor grade in breast with higher apoptotic character [12]. Moreover, iNOS expression has also been implicated in the sensitization of cancer cells to apoptosis [12]. Due to the fact that if iNOS/NO is heavily relied on their concentration, the discrepancies in these studies are possibly related to the specific levels of iNOS and NO in each specific study.

In this study, we have demonstrated the correlation between iNOS protein expression changes after treatment and the responses to neoadjuvant chemotherapy in TNBC patients. We found that the TNBC patients with attenuated expression of iNOS after treatment showed better responses to neoadjuvant chemotherapy than other patients. After that, we confirmed that TNBC cell line with relevant high levels iNOS and NO were less sensitive to apoptosis-induced chemotherapy. Moreover, our data suggested that interfering iNOS or downregulating NO levels in TNBC might have potential to improve the efficacy of platinum-based neoadjuvant chemotherapy.

Above all, our current data suggested the feasibility of treatment of TNBC combining targeted iNOS/NO inhibition and cytotoxic platinum-based chemotherapy. There are several potential mechanisms for iNOS inhibition affecting the resistance of cancer cells to apoptosis, which include interference with PI3K/AKT-mediated overexpression of surviving, or release of caspase activation, or stabilization of Bcl-2 [12]. However, the mechanism and correlation of iNOS and the efficacy of platinum-based neoadjuvant chemotherapy in TNBC needed further investigation.

## Conclusions

The present study found that attenuated iNOS expression after platinum-based neoadjuvant chemotherapy might be a new predictor for triple-negative breast cancer, and iNOS/NO might be a new target of combination. However, the underlying mechanisms of iNOS/NO’s involvement are still unclear. Hence the correlation of iNOS and the efficacy of platinum-based neoadjuvant chemotherapy in TNBC needed further investigation.

## Supporting Information

S1 Fig The original uncropped and unadjusted blots of Fig 2. (TIF)

S2 Fig The original uncropped and unadjusted blots of Fig 5. (TIF)
