# Supplementary figures and images for: Clinical Implications of iNOS Levels in Triple-Negative Breast Cancer Responding to Neoadjuvant Chemotherapy
Source: PLoS One. 2015 Jul 21;10(7):e0130286. doi: 10.1371/journal.pone.0130286 (PMC4510059; doi:10.1371/journal.pone.0130286)

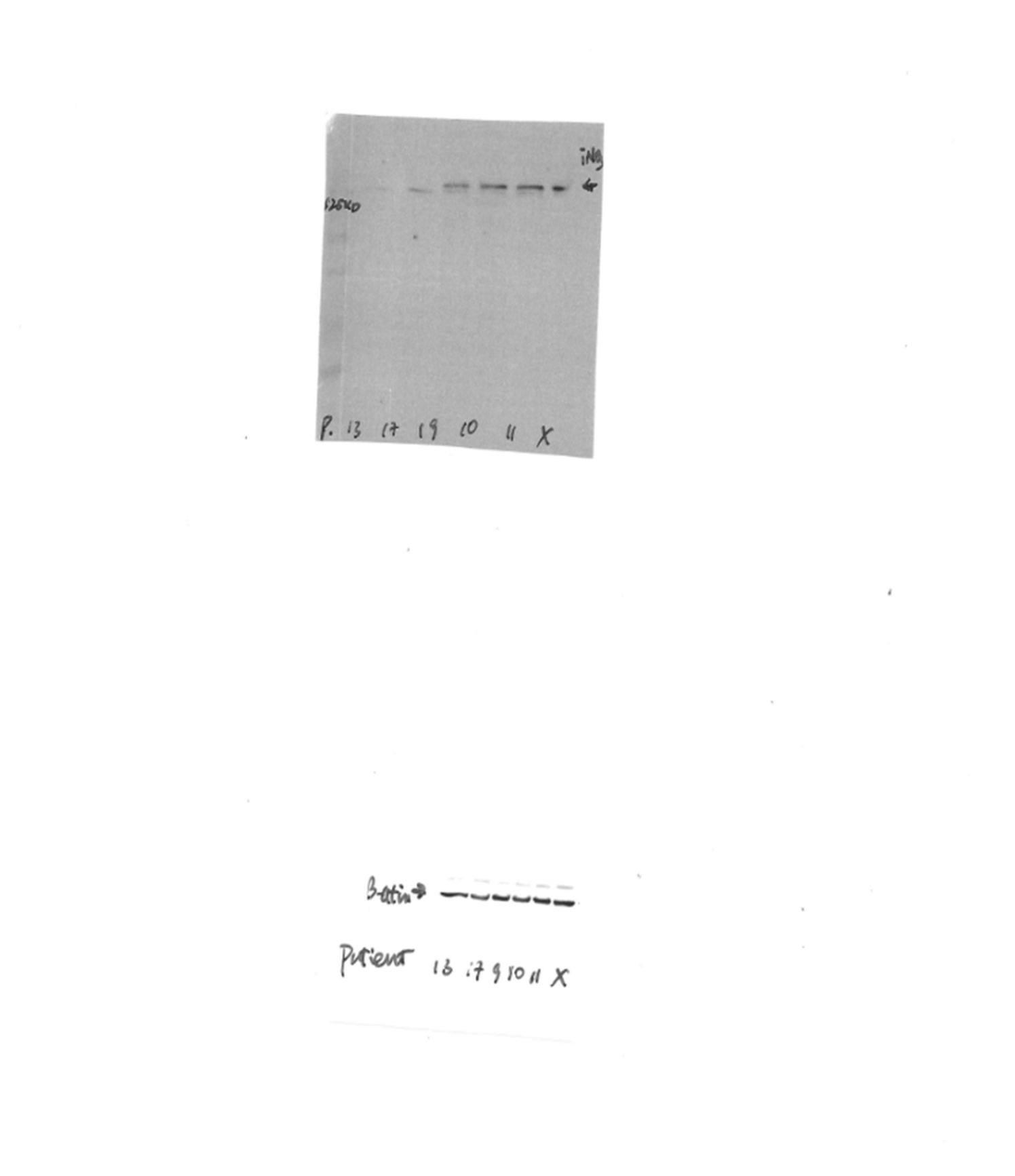

Supplement: S1 Fig — (TIF) [file pone.0130286.s001.tif]

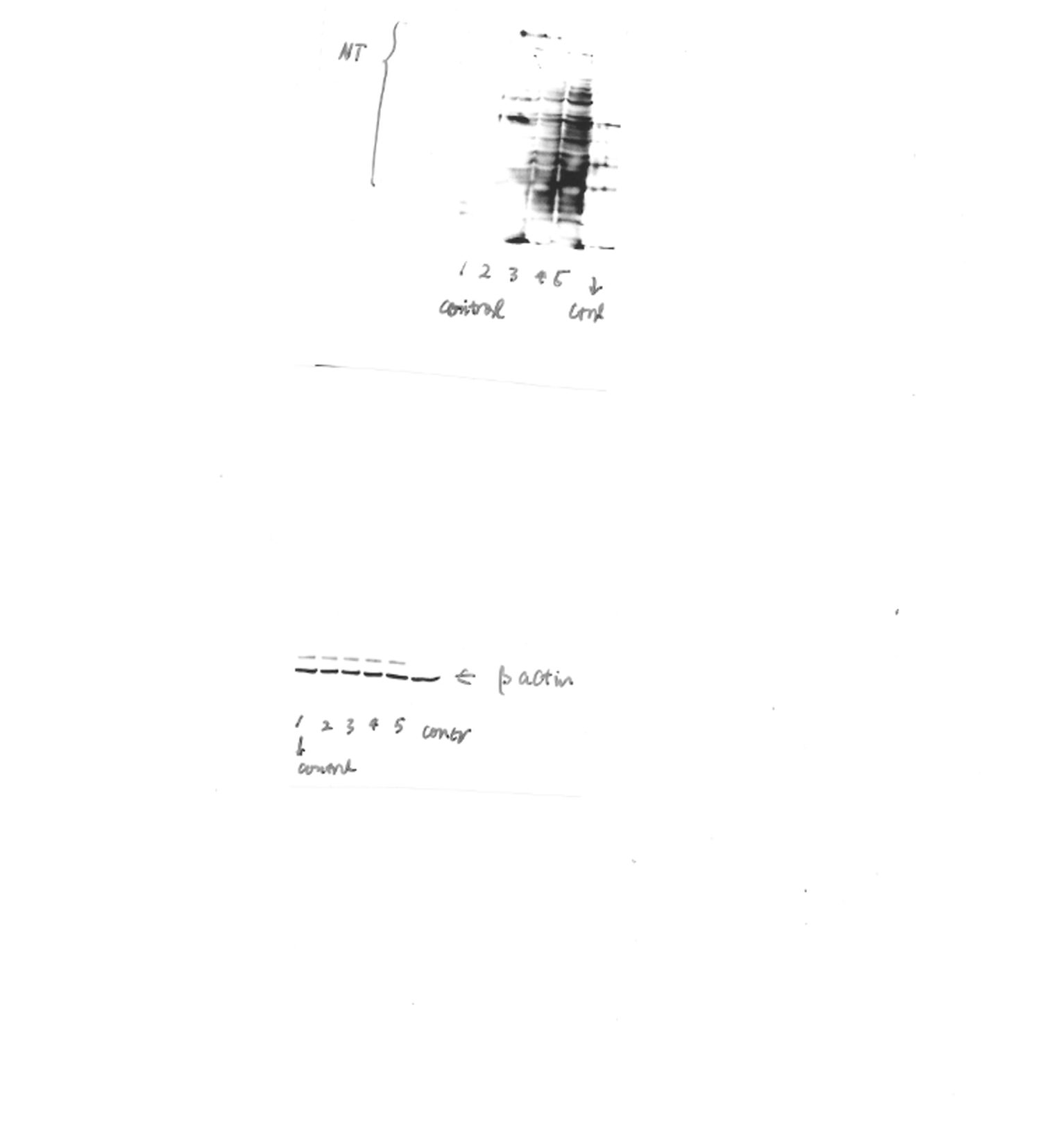

Supplement: S2 Fig — (TIF) [file pone.0130286.s002.tif]
